# The discriminative power of the EuroQol visual analog scale is sensitive to survey language in Singapore

**DOI:** 10.1186/1477-7525-10-32

**Published:** 2012-03-20

**Authors:** Nan Luo, Sheng-Qun Cang, Hui-Min Joanne Quah, Choon-How How, Ee Guan Tay

**Affiliations:** 1 Saw Swee Hock School of Public Health, National University of Singapore, Singapore; 2 Intervet Inc., Summit, NJ, USA; 3 SingHealth Polyclinics, Singapore

**Keywords:** Visual analog scale, EQ-5D-3L, Known-groups validity

## Abstract

**Background:**

Existing evidence for validity of the visual analog scale of the EQ-5D-3L questionnaire (EQ-VAS) is weak in Chinese-speaking respondents in Singapore. We therefore investigated the validity of the Chinese (Singapore) version of EQ-VAS in patients with diabetes.

**Methods:**

In a cross-sectional survey, patients with type 2 diabetes seen in a primary care facility completed an identical Chinese or English questionnaire containing the EQ-5D-3L and questions assessing other health and disease-related characteristics. Convergent and known-groups validity of the EQ-VAS was examined for Chinese- and English-speaking respondents separately.

**Results:**

The EQ-VAS was correlated with the EQ-5D-3L health index and a 5-point Likert-type scale for assessing global health in both Chinese-speaking (N = 335) and English-speaking respondents (N = 298), suggesting convergent validity. The mean EQ-VAS scores differed between English-speaking patients with differing duration of diabetes (< 10 years versus ≥ 10 years), comorbidity status (absence versus presence), and complications of diabetes (absence versus presence), providing evidence for known-groups validity. However, the EQ-VAS scores for Chinese-speaking respondents known to differ in these characteristics were similar, even among subgroups of relatively younger patients or those with formal school education.

**Conclusions:**

Chinese- and English-speaking Singaporeans respond differently to the EQ-VAS. The Chinese version of EQ-VAS appears less sensitive than its English version for measuring global health in patient populations in Singapore.

## Introduction

The visual analog scale (VAS) in the EQ-5D-3L self-report questionnaire [1] is a single-item measure of global health that has demonstrated satisfactory psychometric properties in many populations [2-5]. However, the Chinese version of the EQ-VAS exhibited weak construct validity in Singapore, a multi-ethnic urban country in South-East Asia. In two previous studies in Singapore [6,7], expected associations between the EQ-VAS and other health or clinical measures were not observed among patients with rheumatic or Parkinson' disease who completed the Chinese EQ-5D-3L questionnaire; in contrast, the English version of the EQ-VAS showed good construct validity in the same studies [7,8]. Hence, validity of the EQ-VAS among Chinese-speaking Singaporeans warrants further investigation.

The purpose of the present study was to investigate the construct validity of the EQ-VAS in Chinese-speaking patients with type 2 diabetes using data collected in a health survey of such patients in Singapore. Performance of the English version of the EQ-VAS was also assessed and served as a reference in this study.

## Methods

### Patients and procedures

Outpatients with type 2 diabetes visiting one of the 8 SingHealth Polyclinics over the period of the 6^th ^to 12^th ^January 2009 were recruited for this study using a systematic sampling method. Inclusion criteria were age of 21 years or older, a diagnosis of type 2 diabetes mellitus, and ability to communicate and give informed consent.

All patients going for HbA1c test were assessed for eligibility by trained year-3 medical students. Consenting patients were asked to complete a survey form in the waiting area of the clinics containing the EQ-5D-3L questionnaire, a question for self-assessment of global health, and questions assessing demographic, clinical, and health characteristics. Identical English and Chinese questionnaires were prepared for patients to choose at their own preference. Patients could choose to complete the questionnaire by themselves or through an interviewer.

### Outcome measures

The EQ-5D-3L questionnaire has two pages. Page one is for respondents to report whether they have no, moderate, or extreme problems in mobility, self-care, usual activities, pain/discomfort, and anxiety/depression on the day of survey. An index score ranging from -0.594 to 1.0 (0 = dead; 1.0 = full health) can be calculated from the answers to represent the value of a respondent's health status [9]. The second page is the EQ-VAS for respondents to assess their 'own health state today'. It is a hash-marked, vertical VAS numbered with 0, 10, 20, 30,..., 80, 90, 100 from bottom (0) to top (100). The labels of 'worst/best imaginable health state' are attached to the bottom and top of the scale, respectively.

The question for self-assessment of global health was phrased as 'In general, how would you say your health is?' The response options were 'excellent', 'very good', 'good', 'fair', and 'poor'.

### Data analysis

Convergent validity of the EQ-VAS was assessed according to its correlation with the EQ-5D-3L index and self-assessment of global health. Known-groups validity was evaluated by comparing subgroups of patients known to differ in health status [10]. We hypothesized that the EQ-VAS score would be lower in patients known to have 'worse' health than those had 'better' health. The known groups were defined according to body mass index (non-obese versus obese), duration of diabetes (< 10 years versus 10 or more years), diabetes-related complications (absence versus presence), and comorbid chronic conditions (absence versus presence). Data collected from Chinese and English questionnaires were analyzed separately to assess the validity of both versions of the EQ-VAS. Additionally, in order to examine the possible effects of age and education on the known-groups validity of the Chinese VAS, we assessed the above-mentioned known groups for younger (defined as age < 70 years) and older (defined as age ≥ 70 years) patients separately and for patients with no formal education and those with formal education separately. We hypothesized that the EQ-VAS would be more discriminative among patients of younger age and those with formal education.

Differences in EQ-VAS scores between known-groups were quantified using linear regression models. Socio-demographic characteristics such as age, gender, ethnicity, employment status, education, and survey mode (interviewer-administration versus self-completion), whenever appropriate, were included into the models as independent variables to adjust for their effects on the EQ-VAS score. All statistical tests were two-sided and performed with SAS for Windows (Version 9.2, SAS Institute INC., Cary NC, USA).

## Results

A total of 335 and 298 participants completed the survey in Chinese and English, respectively. Demographic and health characteristics of the study sample are displayed in Table 1. Compared to participants who completed the survey in Chinese, participants completing the survey in English were younger, better educated, and more likely to be females and employed. Accordingly, more Chinese- than English-speaking patients reported one or more comorbidities (80.9% versus 70.8%, p = 0.003) and rated their health as 'fair' or 'poor' (38.8% versus 27.6%, p = 0.003). The majority of patients chose to complete the survey through an interviewer, although a larger proportion of English-speaking patients than Chinese-speaking patients completed the survey by themselves (29.2% versus 5.1%, p < 0.001).

**Table 1:** Characteristics of Patients

	Patients completing the Chinese EQ-5D-3L (N = 335)	Patients completing the English EQ-5D-3L (N = 298)	P value
Age at survey, mean(SD)	65.9 (9.0)	59.2 (10.8)	< 0.001
Male, N (%)	144 (43.1)	157 (53.2)	0.011
Ethnicity, N (%)			
Chinese	335 (100)	166 (56.1)	< 0.001
Malay/India/other	0 (0)	130 (43.9)	
Employment status, N (%)			
Employed	99 (29.6)	140 (47.3)	< 0.001
Retired	139 (41.5)	86 (29.1)	
Housekeeper/unemployed	97 (28.9)	70 (23.6)	
Education attainment, N (%)			
No formal qualifications	134 (40.4)	31 (10.5)	< 0.001
Primary school education	121 (36.4)	59 (20.1)	
Secondary school education	61 (18.4)	117 (39.8)	
Tertiary education	16 (4.8)	87 (29.6)	
BMI category			
Non-obese (BMI < 30), N (%)	280 (87.0)	233 (81.2)	0.122
Obese (BMI ≥ 30), N (%)	42 (13.0)	54 (18.8)	
Duration of diabetes			
< 10 years, N (%)	180 (45.9)	168 (56.9)	0.466
≥ 10 years, N (%)	153 (54.1)	127 (43.1)	
Presence of 1 or more diabetes-related complications, N (%)	144 (43.0)	112 (37.6)	0.167
Presence of 1 or more comorbidities, N (%)	271 (80.9)	211 (70.8)	0.003
Self-reported global heath			
Excellent/very good/good	205 (61.2)	215 (72.4)	0.003
Fair/poor	130 (38.8)	82 (27.6)	
EQ-VAS, mean (SD)	68.9 (16.7)	69.9 (16.8)	0.498
EQ-5D-3L index score, mean(SD)	0.86 (0.18)	0.87 (0.19)	0.519
Administration mode			
Self-completion	17 (5.1)	87 (29.2)	< 0.001
Interviewer-administered	318 (94.9)	211 (70.8)	

Notes: complications were conditions or diseases related to diabetes including stroke, ischemic heart disease, kidney disease, peripheral neuropathy, peripheral vascular disease, and eye disease; comorbidities included cancer, arthritis, hypertension, high blood cholesterol, asthma, lung conditions, liver conditions, mental disorders, urological disease, and ear, nose or throat diseases.

For Chinese-speaking patients, the EQ-VAS was correlated with the EQ-5D-3L index (Spearman's correlation coefficient: 0.27) and self-assessed global health (Spearman's correlation coefficient: -0.51), suggesting convergent validity. However, there was no statistical difference in EQ-VAS scores between subgroups of patients known to differ in BMI, duration of diabetes, complication status, or comorbidity status in both univariate and multivariate analysis, suggesting poor known-groups validity (Table 2). For example, the multiple regression analysis showed that the difference in EQ-VAS score between patients with and without any comorbidity was 1.7 (p > 0.05, *t*-test) after adjusting for socio-demographic status. Subgroup analyses suggested that known-groups validity was not better among patients with formal education than those without formal education (Table 3), or among younger patients than older patients (Table 4).

**Table 2:** Comparison of EQ-VAS Scores between Subgroups with Different Health Status: by Survey Language

	Patients completing the Chinese EQ-5D-3L			Patients completing the English EQ-5D-3L		
	N	Mean (SD)	Adjusted difference* (p-value)	N	Mean (SD)	Adjusted difference* (p-value)
BMI (kg/m^2^)						
< 30	280	69.0 (16.5)	-1.4	233	70.7 (16.4)	3.0
≥ 30	42	70.7 (17.6)	(0.612)	54	67.6 (16.4)	(0.241)
Duration of DM						
< 10 years	180	69.7 (17.7)	1.1	168	72.8 (15.1)	5.8
≥ 10 years	153	68.1 (15.1)	(0.564)	127	66.8 (17.5)	(0.003)
Presence of complications						
No	188	70.7 (17.5)	3.6	184	73.4 (14.2)	7.8
Yes	144	67.0 (15.4)	(0.057)	112	65.0 (18.3)	(< 0.001)
Presence of comorbidities						
No	61	68.4 (17.8)	-1.7	85	75.6 (15.4)	6.8
Yes	271	69.2 (16.4)	(0.493)	211	68.0 (16.3)	(0.002)

Notes: complications were conditions or diseases related to diabetes including stroke, ischemic heart disease, kidney disease, peripheral neuropathy, peripheral vascular disease, and eye disease; comorbidities included cancer, arthritis, hypertension, high blood cholesterol, asthma, lung conditions, liver conditions, mental disorders, urological disease, and ear, nose or throat diseases. * values are regression coefficients in multiple linear regression models in which the effects of administration mode, age, gender, employment status, and education using linear regression models are adjusted for.

**Table 3:** Comparison of EQ-VAS Scores between Subgroups with Different Health Status: by Education Level for Patients Completing the Chinese EQ-5D-3L

	No formal education (N = 134)			With formal education (N = 198)		
	N	Mean (SD)	Adjusted difference* (p-value)	N	Mean (SD)	Adjusted difference* (p-value)
BMI (kg/m^2^)						
< 30	109	69.5 (17.1)	1.4	170	68.8 (16.1)	-4.1
≥ 30	18	66.9 (18.6)	(0.754)	24	73.5 (16.7)	(0.257)
Duration of DM						
< 10 years	65	70.8 (18.0)	4.5	114	69.3 (17.1)	-1.4
≥ 10 years	69	66.4 (16.3)	(0.143)	84	69.5 (15.3)	(0.579)
Presence of complication						
No	74	69.4 (18.9)	1.9	114	71.5 (16.7)	4.0
Yes	60	67.6 (14.9)	(0.538)	84	66.5 (15.3)	(0.104)
Presence of comorbidities						
No	21	63.3 (14.5)	-6.0	40	71.0 (18.9)	0.9
Yes	113	69.5 (17.5)	(0.166)	158	68.9 (15.6)	(0.771)

Notes: complications were conditions or diseases related to diabetes including stroke, ischemic heart disease, kidney disease, peripheral neuropathy, peripheral vascular disease, and eye disease; comorbidities included cancer, arthritis, hypertension, high blood cholesterol, asthma, lung conditions, liver conditions, mental disorders, urological disease, and ear, nose or throat diseases. * values are regression coefficients in multiple linear regression models in which the effects of administration mode, age, gender, employment status, and education using linear regression models are adjusted for.

**Table 4:** Comparison of EQ-VAS Scores between Subgroups with Different Health Status: by Age Group for Patients Completing the Chinese EQ-5D-3L

		< 70 years (N = 211)			70 years or older (N = 124)	
	N	Mean (SD)	Adjusted difference* (p-value)	N	Mean (SD)	Adjusted difference* (p-value)
BMI (kg/m^2^)						
< 30	173	70.2 (16.7)	0.0	107	67.1 (16.0)	-5.1
≥ 30	32	70.8 (16.7)	(0.997)	10	70.4 (21.3)	(0.354)
Duration of DM						
< 10 years	130	70.6 (17.6)	1.3	50	67.4 (17.0)	0.4
≥ 10 years	81	68.9 (15.3)	(0.584)	72	67.2 (16.4)	(0.897)
Presence of complication						
No	137	71.3 (17.3)	3.3	51	69.0 (18.4)	1.7
Yes	73	67.8 (15.3)	(0.185)	71	66.1 (15.1)	(0.589)
Presence of comorbidities						
No	47	68.0 (18.7)	-3.4	14	69.6 (15.1)	-0.2
Yes	163	70.7 (16.0)	(0.239)	108	67.0 (16.8)	(0.964)

Notes: complications were conditions or diseases related to diabetes including stroke, ischemic heart disease, kidney disease, peripheral neuropathy, peripheral vascular disease, and eye disease; comorbidities included cancer, arthritis, hypertension, high blood cholesterol, asthma, lung conditions, liver conditions, mental disorders, urological disease, and ear, nose or throat diseases. * values are regression coefficients in multiple linear regression models in which the effects of administration mode, age, gender, employment status, and education using linear regression models are adjusted for.

In contrast, the EQ-VAS demonstrated both convergent and known-groups validity among patients who elected to complete the survey in English. Spearman's correlation coefficient was 0.31 between the EQ-VAS and EQ-5D-3L index and -0.56 between the EQ-VAS and self-assessed global health. Patients with 1 or more diabetes-related complications or comorbidities had lower EQ-VAS scores than those without such conditions, and patients who had diabetes for < 10 years had higher EQ-VAS scores than those who had diabetes for 10 or more years. Those differences were statistically significant even after controlling for the effect of socio-demographic status in the multiple regression models (Table 2). It was also as hypothesized that non-obese patients had higher EQ-VAS scores than obese patients, although the difference was not statistically significant. It should be noted that the magnitude of the mean differences between the comparison groups was not larger (range: 3.0 to 7.8)

## Discussion

In the present study, the EQ-VAS exhibited poor known-groups validity among Chinese-speaking patients with diabetes, although convergent validity was demonstrated by correlations between the EQ-VAS and two other measures of overall health. In contrast, both convergent and known-groups validity were observed for the English EQ-VAS. Similar results were also observed for the EQ-VAS in patients with rheumatic diseases [6], Parkinson's disease [7], and breast cancer (Yin-Bun Cheung, personal communication). Therefore, it appears that the Chinese EQ-VAS is not a sensitive measure for self-assessment of overall health in Singaporean patient populations. To the best of our knowledge, no previous studies questioned the sensitivity of the EQ-VAS in specific or the visual analog scale in general.

Our finding from the present study has some important implications. First, our study highlighted the importance of psychometric testing for health-status instruments. Good measurement properties of an instrument in one population may not necessarily be generalized to other populations especially those multi-cultural populations. This is true even for widely used simple instruments such as the VAS. Herdman et al. pointed out that measurement equivalence across language versions should be examined in cross-cultural application of health-related quality of life instruments [11]. Second, our study suggested that the EQ-VAS is not a sensitive measure for Chinese-speaking patients in Singapore. Although being a valid measure, the EQ-VAS may not be able to detect true differences between groups when such differences are small. When a measure for overall health is needed for this population, the EQ-5D-3L index may a better choices as it demonstrated better known-groups validity in the present study (see Table 5). Third, we can reasonably suspect that other variants of the VAS used in clinical research or practice in Singapore may suffer from similar problems when they are applied to Chinese-speaking patients. Since no previous studies have looked into the psychometric properties of other VAS variants in Singapore, investigators should be cautious when interpreting data collected from Chinese-speaking patients using such scales.

**Table 5:** Comparison of EQ-5D-3L Index Scores between Subgroups with Different Health Status: by Survey Language

	Patients completing the Chinese EQ-5D-3L			Patients completing the English EQ-5D-3L		
	N	Mean (SD)	Adjusted difference* (p-value)	N	Mean (SD)	Adjusted difference* (p-value)
BMI (kg/m^2^)						
< 30	280	0.867 (0.174)	0.020	231	0.892 (0.161)	0.080
≥ 30	41	0.849 (0.207)	(0.501)	54	0.799 (0.234)	(0.003)
Duration of DM						
< 10 years	179	0.882 (0.139)	0.034	168	0.897 (0.161)	0.043
≥ 10 years	153	0.836 0.209)	(0.090)	125	0.843 (0.199)	(0.039)
Presence of complications						
No	187	0.897 (0.138)	0.073	183	0.917 (0.113)	0.102
Yes	144	0.814 (0.208)	(< 0.001)	111	0.803 (0.239)	(< 0.001)
Presence of comorbidities						
No	60	0.889 (0.130)	0.011	85	0.926 (0.148)	0.056
Yes	271	0.855 (0.185)	(0.685)	209	0.852 (0.187)	(0.015)

Notes: complications were conditions or diseases related to diabetes including stroke, ischemic heart disease, kidney disease, peripheral neuropathy, peripheral vascular disease, and eye disease; comorbidities included cancer, arthritis, hypertension, high blood cholesterol, asthma, lung conditions, liver conditions, mental disorders, urological disease, and ear, nose or throat diseases. * values are regression coefficients in multiple linear regression models in which the effects of administration mode, age, gender, employment status, and education using linear regression models are adjusted for.

It is intriguing why the EQ-VAS performed differently among Chinese- and English-speaking Singaporeans. We thought older age and poor education might be the reasons as those were the main differences between Chinese- and English-speaking patients. We speculated that some older patients or poorly educated patients might not know how to use the EQ-VAS for self-rating because of age-related cognitive impairment or poor numeracy, respective. However, our results did not support this hypothesis; the insensitivity of EQ-VAS to different health status was not associated with education or age (Tables 3 and 4). Although determining the real reason for the observed results is beyond the scope of the present study, the possible reasons should be related to different response styles of the respondents. It may be that Chinese speakers in Singapore have some idiosyncratic response style such that relatively healthy Chinese-speaking patients score their own health lower than their English counterparts on the VAS. Chinese philosophies such as Middle Way [12] may make practitioners avoid using high or low VAS scores to describe their own health. Chinese people might be reluctant to say their health is very good because they are afraid that God may punish them for not being humble [13]. However, we cannot rule out the possibility that the poor performance of the Chinese EQ-VAS was due to the suboptimal translation of the instructions and anchor labels of the instrument. Future studies using qualitative research methods such as focus group discussion should be conducted to elicit the causes for the poor sensitivity to difference of the Chinese version of the EQ-VAS.

There were some limitations in our study. First, all data used in our study were self-reported data. Chinese-speaking patients might have reported less accurate information on complication or comorbidity profiles than English-speaking patients because they were older. If this was the case, the known-groups validity of the Chinese EQ-VAS would have been underestimated in this study. Second, the internal validity of our results might have been affected by respondents' self-selection of survey languages. It is possible that those bilingual respondents who choose the Chinese questionnaires happened to have different response style. Ideally, bilingual respondents were identified and randomized to complete the survey in English or Chinese. Third, the finding of our study only has limited external validity. Since our finding is purely based on patients with diabetes, it may not be generalized to all patient populations in Singapore. Nevertheless, similar results were also observed for rheumatic diseases, Parkinson's disease, and breast cancer. Additionally, our study may not be generalized to other Chinese-speaking populations such as Chinese in mainland China. A recent cross-sectional study of patients with diabetes in China found that the EQ-VAS score was associated with duration of diabetes and microvascular complications but not with BMI or macrovascular complications [14]. In spite of limited generalizability, our study demonstrated the necessity of cross-cultural validation of even simple health-status measures such as the VAS.

In conclusion, compared to its English counterpart, the EQ-VAS appears less sensitive to different health status in Chinese-speaking patients with type 2 diabetes in Singapore. Future studies using qualitative research methods are needed to ascertain the underlying reasons.

## Abbreviations

EQ-VAS: EQ-5D Visual Analog Scale; BMI: Body Mass Index.

## Competing interests

The authors declare that they have no competing interests.

## Authors' contributions

NL conceptualized the paper, co-wrote the manuscript, and verified the data analysis. SQC analyzed the data and wrote the first draft of the paper. JHQ, CHH, and EGT contributed to data interpretation and manuscript refinement. All authors have read and approved the final version of the manuscript.
